# 

**Published:** 1858-06

**Authors:** 


					﻿PUBLISHED MONTHLY. AT S3 PER ANNUM IN ADVANCE.
x
©
fl
©
s
©
s
a
a
©
©
*3
&
a
a
I
ci
©
r»
©
®
fl
©
©
©
eo
X
©
g
©
©
©
►
©
P
d
©
d
x
fl
©
O
rH
x
©
g
o
©
5.
>S
s
I
«
<
h
x
©
a.
ATLANTA
iiuiini. mi siii.ii'il
s
>0fIlli.
EDITED BY
JOSEPH P. LOGAN, M. D.,
Professor of Physiology axd General Pathology in the Atianta Medical College.
AND
W. F. WESTMORELAND, M. D.,
Professor of the Principles and Practice of Surgery in the Atlanta Medical College,
Pax et scientia, sed veritas sine timore
Vol. in.	JUNE, 1858.	No. X.
ATLANTA, GEORGIA:
PRINTED BY G- P- EDDY & CO.
18 5 8.
EACH NUMBER CONTAINS SIXTY-FOUR PAGES.
©
X
B
©
X
©
X
fl
ft
ft
ft
5
B
5
9
ft
©
•5
*7
r
©
©
«•
a*
M
5.
i
S’
t?
X
S
©
S’
o
$
sr
ft
fl
5
©
a
s
>
X
J?
X
ft
fl-
fl
?
ft
ft*
©
a
0
ft
X
				

